# Scaling and kinematics optimisation of the scapula and thorax in upper limb musculoskeletal models

**DOI:** 10.1016/j.jbiomech.2014.05.015

**Published:** 2014-08-22

**Authors:** Joe A.I. Prinold, Anthony M.J. Bull

**Affiliations:** Department of Bioengineering, Imperial College London, London SW7 2AZ, UK

**Keywords:** Biomechanics, Subject specific, Modelling, Shoulder, Conoid ligament, Pull-up

## Abstract

Accurate representation of individual scapula kinematics and subject geometries is vital in musculoskeletal models applied to upper limb pathology and performance. In applying individual kinematics to a model׳s cadaveric geometry, model constraints are commonly prescriptive. These rely on thorax scaling to effectively define the scapula׳s path but do not consider the area underneath the scapula in scaling, and assume a fixed conoid ligament length. These constraints may not allow continuous solutions or close agreement with directly measured kinematics.

A novel method is presented to scale the thorax based on palpated scapula landmarks. The scapula and clavicle kinematics are optimised with the constraint that the scapula medial border does not penetrate the thorax. Conoid ligament length is not used as a constraint. This method is simulated in the UK National Shoulder Model and compared to four other methods, including the standard technique, during three pull-up techniques (*n*=11). These are high-performance activities covering a large range of motion.

Model solutions without substantial jumps in the joint kinematics data were improved from 23% of trials with the standard method, to 100% of trials with the new method. Agreement with measured kinematics was significantly improved (more than 10° closer at *p*<0.001) when compared to standard methods. The removal of the conoid ligament constraint and the novel thorax scaling correction factor were shown to be key. Separation of the medial border of the scapula from the thorax was large, although this may be physiologically correct due to the high loads and high arm elevation angles.

## 1 Introduction

Scaling of musculoskeletal models is important to accurately represent inter-segmental joint moments. Equal scaling of all segments improves accuracy of force predictions in dynamic tasks, but has little effect in static-loaded tasks (Nikooyan et al., 2010): where external loads are important. Once a model׳s geometry has been scaled and measured kinematics supplied as inputs, it is necessary to test that the model remains physiological; solid structures must not overlap and close agreement with measured kinematics is important: within the measurement errors of dynamic measurement techniques (e.g. Shaheen et al., 2011a).

Commonly, constrained optimisation of measured kinematics is used (e.g. Dickerson et al., 2007). These methods constrain the medial border of the scapula to a fixed offset from the thorax surface, the conoid ligament to a fixed length and do not scale the generic model (Nikooyan et al., 2011). These constraints define the scapula׳s path based on thorax scaling methods that do not currently consider the area underneath the scapula. This method does not regularly provide continuous solutions (Martelli et al., 2008), and may result in large differences between measured and optimised angles (Bolsterlee et al., 2012).

According to open-MRI (Izadpanah et al., 2012) and CT (Seo et al., 2012) studies, the conoid ligament is not a fixed length during arm abduction. The conoid ligament׳s length and attachments vary by up to 25% between subjects (Harris et al., 2001, Takase, 2010), and may scale with clavicle length (Rios et al., 2007). It is therefore theorised that relaxation of the standard kinematics optimisation constraints and redefinition of thorax scaling will allow more robust scalable simulations that maintain close agreement with measured kinematics.

## 2 Methodology

### 2.1 Materials and data

**Fig. 1:**
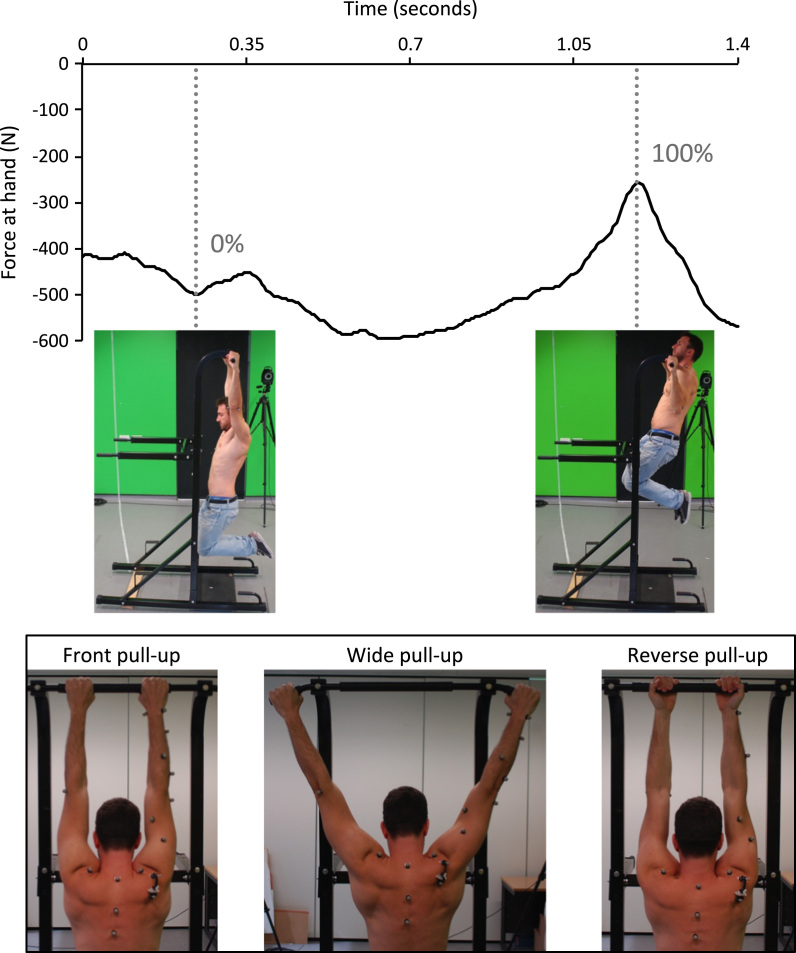
Upper portion illustrates the normalisation used for the pull-up tasks: the left side shows 0% of the wide pull-up motion and the right side shows 100% of the wide pull-up motion. The trace is the force at the hand in a representative trial. The lower portion of the figure illustrates the hand position in the three pull-up tasks (viewed from behind the subject).

Eleven consenting subjects participated (mean age: 26±3 years). Three pull-up types were performed: front, wide and reverse (Fig. 1). Subjects were instructed to perform a maximal upward movement: from hanging with arms straight to full elevation (chin above the bar). Mean activity time was 1.2 s, with ranges of: 0.8–2.2, 0.9–1.6 and 0.9–1.8 s for the front, wide and reverse pull-ups respectively. Legs were kept in a fixed position with posterior-facing heels (Fig. 1). Pull-ups dynamically cover large ranges of motion: 23–126° humerothoracic elevation and −56−10° humerothoracic axial rotation.

The UK National Shoulder Model (UKNSM; Charlton and Johnson, 2006) is the musculoskeletal model used for the simulations: accepting marker positions to calculate local ISB-recommended coordinate systems and Euler rotations (Wu et al., 2002) to then apply inverse dynamics and static optimisation. A nine-camera Vicon motion capture system (200 Hz) recorded marker positions.

### 2.2 Scaling

Clavicle and scapula segments were homogeneously scaled based on relative segment lengths between model and subject. Clavicle scaling used sternoclavicular (SC) to acromioclavicular (AC) joint distance and scapula scaling AC to scapula inferior angle (AI) distance.

An ellipse represented the scapulothoracic gliding-plane (STGP): described by Charlton (2003). In this study a novel technique is described to improve the thorax STGP scaling. Firstly, a homogeneous scaling factor was defined for the STGP ellipse based on subject height (Eq. (1)). Static measurements of bony landmarks were taken: (1) at rest with the arms by the side, (2) arms horizontal at 45° to the coronal plane and (3) arms at subject׳s maximal elevation. The following landmarks were measured in these three static poses:

• Acromial angle, trigonum spinae (TS) and AI (measured using the scapula Palpator; Shaheen et al., 2011b).
• SC, jugular notch, xiphoid process, 7th cervical vertebra and 8th thoracic vertebra (measured using skin-fixed markers).
• AC joint measured relative to the scapula Tracker, digitised at 90° elevation and 45° horizontal abduction (Prinold et al., 2011).
  (1) $$T_{h}=\frac{\text{height}\;\;\mathrm{of}\;\mathrm{subject}}{1.80}$$
  where *T*_*h*_ is the initial homogeneous scaling factor for the thorax ellipse. The cadaver׳s height, used for the original UKNSM, was 1.80 m.

An optimisation procedure was then used to define the thorax ellipse size and position. The constraint applied to the optimiser was that the scapula medial border (TS and AI) could not fall within the STGP ellipse. The optimiser objective function kept the non-homogeneous scaling factors as close as possible to the original homogeneous scaling factor in a least squares sense (Eq. (2)). The new non-homogeneous scaling factors (*T*_*x,y,z*_) were then applied to the size and centre position of the STGP ellipse and the thorax-body ellipse for the pull-up simulations.

(2) $$\begin{array}{l} \min\;[{\left(T_{x}-T_{h}\right)}^{2}+{\left(T_{y}-T_{h}\right)}^{2}+{\left(T_{z}-T_{h}\right)}^{2}]\\ \mathrm{such}\;\mathrm{that}:\\ \left[{\left(\frac{AI_{x,i}-M_{x}}{A_{x}}\right)}^{2}+{\left(\frac{AI_{y,i}-M_{y}}{A_{y}}\right)}^{2}+{\left(\frac{AI_{z,i}-M_{z}}{A_{z}}\right)}^{2}-1\right]\geq 0\\ \left[{\left(\frac{TS_{x,i}-M_{x}}{A_{x}}\right)}^{2}+{\left(\frac{TS_{y,i}-M_{y}}{A_{y}}\right)}^{2}+{\left(\frac{TS_{z,i}-M_{z}}{A_{z}}\right)}^{2}-1\right]\geq 0 \end{array}$$

Here *T*_*x*_, *T*_*y*_, *T*_*z*_ are the non-homogeneous thorax scaling parameters being optimised; *i* indicates each of the 3 arm positions; *AI*_*x,i*_*, AI*_*y,i*_, *AI*_*z,i*_ are the position of AI in trial *i*; *TS*_*x,i*_, *TS*_*y,i*_, *TS*_*z,i*_ are the position of TS in trial *i*; *M*_*x*_, *M*_*y*_, *M*_*z*_ are the centre of the thorax ellipse; *A*_*x*_, *A*_*y*_, *A*_*z*_ are the vectors corresponding to axes lengths. The ellipse thickness of 10 mm is included here through addition to the axes lengths in each dimension.

Eq. (2) is the optimisation and constraints used to find the thorax ellipses scaling factors.

### 2.3 Kinematics optimisation

The fixed closed chain (FCC) method optimises the scapula and clavicle kinematics to reduce the least squares difference to the measured scapula and clavicle kinematics. Meanwhile the scapula medial border is constrained to stay 10 mm from the STGP ellipse and the conoid ligament constrained to a fixed length (Eq. (3); van der Helm, 1994).

The partially closed chain (PCC) method also optimises the scapula and clavicle kinematics to reduce the least squares difference to the measured kinematics. However, the scapula medial border is only constrained to not penetrate the STGP ellipse and the conoid ligament length is not used as a constraint:

(3) $$\;\min\;[2({\left(Sx_{m}-Sx_{0}\right)}^{2}+{\left(Sy_{m}-Sy_{0}\right)}^{2}+{\left(Sz_{m}-Sz_{0}\right)}^{2})+(0.75{\left(Cx_{m}-Cx_{0}\right)}^{2}\;+1({\left(Cy_{m}-Cy_{0}\right)}^{2}+{\left(Cz_{m}-Cz_{0}\right)}^{2}))]\mathrm{for}\;\mathrm{PCC}\;\mathrm{method}\;\mathrm{such}\;\text{that}:\left[{\left(\frac{AI_{x}-M_{x}}{A_{x}}\right)}^{2}+{\left(\frac{AI_{y}-M_{y}}{A_{y}}\right)}^{2}+{\left(\frac{AI_{z}-M_{z}}{A_{z}}\right)}^{2}-1\right]\geq 0\left[{\left(\frac{TS_{x}-M_{x}}{A_{x}}\right)}^{2}+{\left(\frac{TS_{y}-M_{y}}{A_{y}}\right)}^{2}+{\left(\frac{TS_{z}-M_{z}}{A_{z}}\right)}^{2}-1\right]\geq 0\mathrm{or}\;\mathrm{for}\;\mathrm{FCC}\;\mathrm{method}\;\mathrm{such}\;\text{that}:\left[{\left(\frac{AI_{x}-M_{x}}{A_{x}}\right)}^{2}+{\left(\frac{AI_{y}-M_{y}}{A_{y}}\right)}^{2}+{\left(\frac{AI_{z}-M_{z}}{A_{z}}\right)}^{2}-1\right]=0\left[{\left(\frac{TS_{x}-M_{x}}{A_{x}}\right)}^{2}+{\left(\frac{TS_{y}-M_{y}}{A_{y}}\right)}^{2}+{\left(\frac{TS_{z}-M_{z}}{A_{z}}\right)}^{2}-1\right]=0L_{rest}^{con}-L^{con}=0$$

The scapula angles; *Sx*, *Sy*, *Sz* are the scapulothoracic Euler angles being optimised. The clavicle angles; *Cx*, *Cy*, *Cz* are the claviculothoracic Euler angles being optimised. *L*^*con*^ is the length of the conoid ligament; computed based on the optimised scapulothoracic and clavicle angles. *AI*_*x*_, *AI*_*y*_, *AI*_*z*_ are the coordinates of AI and *TS*_*x*_, *TS*_*y*_, *TS*_*z*_ are the coordinates of TS; both of which are computed based on the optimised scapulothoracic and clavicle angles.

The constant values are the measured scapulothoracic Euler rotations (*Sx*_*m*_, *Sy*_*m*_, *Sz*_*m*_), the claviculothoracic Euler angles (*Cx*_*m*_*, Cy*_*m*_*, Cz*_*m*_), the rest length of the conoid ligament ($L_{rest}^{con}$), the coordinates of the centre of the scapula gliding-plane ellipse (STGP) after the described thorax scaling procedure has been applied (*M*_*x*_, *M*_*y*_, *M*_*z*_) and the axes lengths of the STGP ellipse after the described thorax scaling procedure has been applied and including an ellipse thickness of 10 mm (*A*_*x*_, *A*_*y*_, *A*_*z*_).

Eq. (3) is the kinematics optimisation objective function with constraints for the described FCC and PCC methods.

Variations of the PCC and FCC methods are tested to understand the effect of each element of the optimisation (Table 1). Clavicle axial rotation was calculated with regression equations in the PCC-based methods (Charlton and Johnson, 2006) and with a minimisation technique in the FCC-based methods (van der Helm, 1994).

**Table 1:** Details of the five kinematics optimisation methods simulated. The scaling methods used are also described. Note that the PCC and FCC methods have been described in detail in Section 2.3; the other three methods are variations of these. ‘Correction factor’ refers to the optimisation-based thorax scaling method described in Section 2.2. ‘Homogeneous scaling based on height’ refers to scaling of the STGP ellipse according to Eq. (1). ‘Segment homogeneous’ refers to homogeneous scaling of each segment individually, as described in Section 2.2.

	**PCC**	**PCC with a fixed conoid length**	**PCC with no STGP correction factor**	**FCC**	**FCC with no conoid length constraint**
*Abbreviation used*	*PCC*	*PCC with con*	*PCC no cf*	*FCC*	*FCC no con*
STGP ellipse scaling	Scaling correction factor used	Scaling correction factor used	Homogeneous scaling based on height	No scaling	No scaling
Other scaling	Segment homogeneous	Segment homogeneous	Segment homogeneous	No scaling	No scaling
TS and AI constraint	Not penetrating STGP ellipse	Not penetrating STGP ellipse	Not penetrating STGP ellipse	Fixed distance from STGP ellipse	Fixed distance from STGP ellipse
Conoid constraint	No constraint	Fixed length	No constraint	Fixed length	No constraint

A sequential quadratic programming algorithm implemented in the fmincon function of MATLAB (v2012b) was used to perform the kinematics optimisation. A first-order-optimality measure based on Karush–Kuhn–Tucker (KKT) conditions was calculated, and used as the optimiser׳s stopping criterion. In frame one of each trial the initial joint angles given to the optimiser were randomly varied five hundred times within physiologically feasible bounds (±22.5°). The most optimal solution to the kinematics optimisation, based on the lowest function value (Eq. (3)), was then found from these five hundred solutions. The most optimal solution was then used as the starting point for the second frame optimisation. For the following frames the previous solution was the starting point to the optimiser.

### 2.4 Data processing

The mean differences to measured rotations were not normally distributed (Shapiro–Wilk). Friedman tests tested for differences across the five optimisation-methods in the three motions (Table 1). A post-hoc Wilcoxon signed-rank test with Holm׳s correction for multiple comparisons identified significant differences between pairs of optimisation methods within each motion.

## 3 Results

The FCC optimisation method leads to solutions with substantial jumps in the data for most subjects. The PCC optimisation method gives data that could be fitted to a continuous function, without substantial jumps, in all trials and subjects (Table 2).

**Table 2:** Percentage of measured pull-up trials containing substantial jumps in the data for the clavicle and scapula rotations during three pull-up motions (*n*=11 for each pull-up technique)

	PCC (%)	PCC con (%)	PCC no cf (%)	FCC (%)	FCC no con (%)
Front	0	3	0	85	52
Wide	0	3	9	73	42
Reverse	0	0	0	73	36

**Fig. 2:**
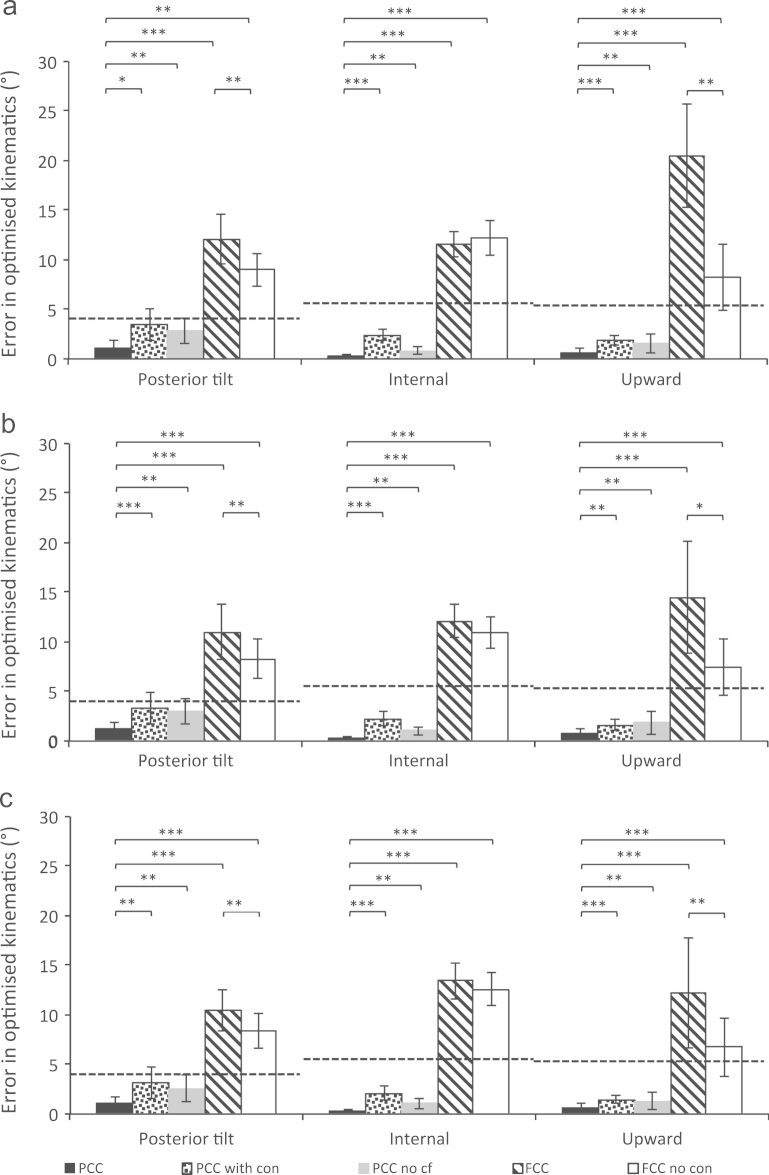
Differences between average optimised scapulothoracic rotations and measured kinematics for (a) front, (b) wide and (c) reverse configuration pull-ups using various scaling and kinematics optimisation strategies (described in Table 1), including the 95% confidence intervals of the differences. Scapulothoracic measurement errors are shown as a dashed horizontal line. These values are the average absolute error plus three SDs (note not RMS) found for each rotation in a previous study (Prinold et al., 2011). The measurement error calculated in upward rotation was 5.6° (compared to 8.4° RMS error in abduction or forward flexion in a bone-pin study: Karduna et al., 2001), the value in internal rotation was 5.8° (compared to 3.8° RMS error in Karduna et al. (2001)) and for posterior tilt 4.9° (compared to 6.2° RMS error in Karduna et al. (2001)). ^⁎^indicates *p*<0.05, ^⁎⁎^*p*<0.01, ^⁎⁎⁎^*p*<0.0001. All trials were included in the statistical analysis. The abbreviations used for the optimisation methods are described in Table 1. Additional simulations using homogeneous scaling of all segments based on segment length (including the thorax) and the FCC method constraints, found average differences across the three pull-up configurations of: 9.0±2.8° (ST posterior tilt), 9.0±2.8° (ST internal) and 9.0±2.8° (ST upward). The same values, with homogeneous scaling of all segments, but without a constrained conoid ligament length were smaller: 4.6±1.9° (ST posterior tilt), 2.2±0.7° (ST internal) and 5.5±2.3° (ST upward).

**Fig. 3:**
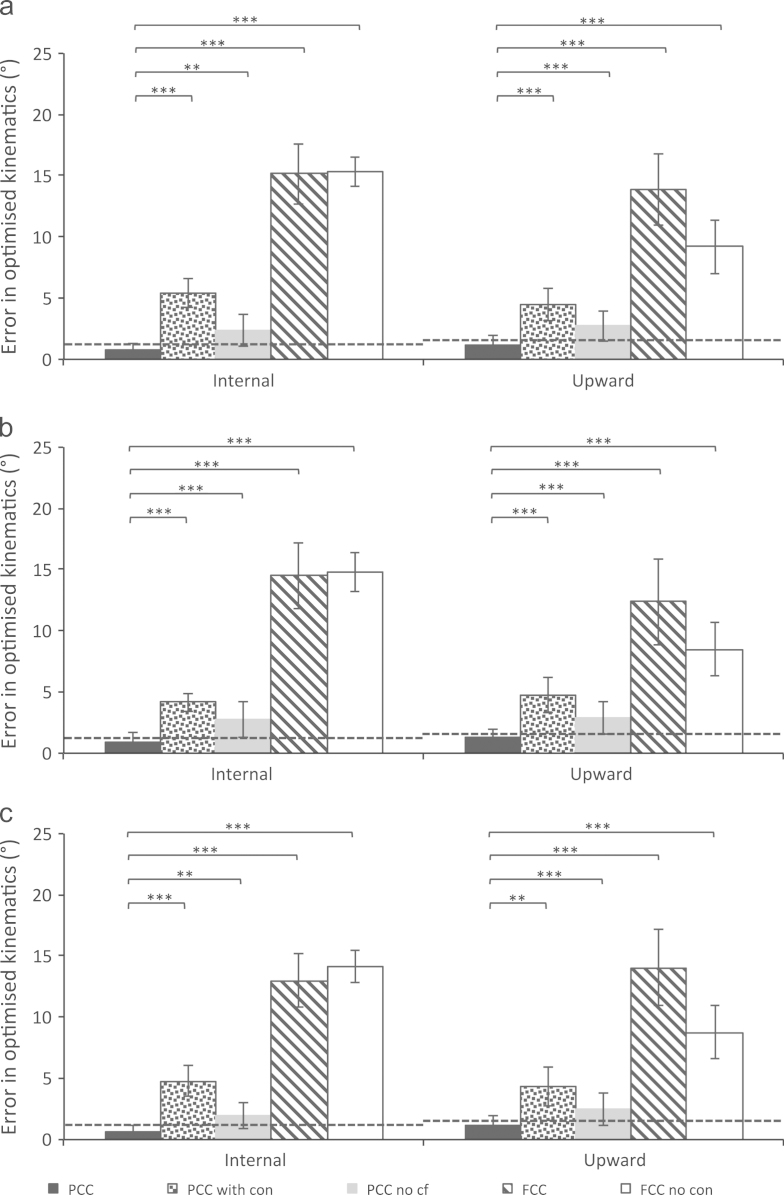
Average clavicle rotation differences to measured kinematics for (a) front, (b) wide and (c) reverse configuration pull-ups using various scaling and kinematics optimisation strategies (described in Table 1), including the 95% confidence intervals of the kinematics. The average RMS clavicle measurement errors found for the Scapula Tracker in a bone pin validation study (Karduna et al., 2001) are included as a dashed horizontal line. These values are 1.2° and 1.6° for clavicle protraction and upward rotation respectively. ^⁎^indicates *p*<0.05, ^⁎⁎^*p*<0.01, ^⁎⁎⁎^*p*<0.0001. Note all trials were included in the statistical analysis. The abbreviations used for the optimisation methods are described in Table 1. Additional simulations using homogeneous scaling of all segments based on segment length (including the thorax) and the FCC method constraints, found average differences across the three pull-up configurations of: 10.9±3.3° (CT internal) and 9.0±2.8° (CT upward). The same values, with homogeneous scaling of all segments, but without a constrained conoid ligament length were smaller: 6.1±2.2° (CT internal) and 7.9±3.2° (CT upward).

The effect of different optimisation parameters are compared through mean differences to measured rotations and 95% confidence intervals (C.I.) of those optimised rotations (Fig. 2, Fig. 3). The PCC optimisation method is closest to the measured values – with significantly smaller errors in many cases. All the other methods fall outside the scapulothoracic measurement errors (Fig. 2).

**Fig. 4:**
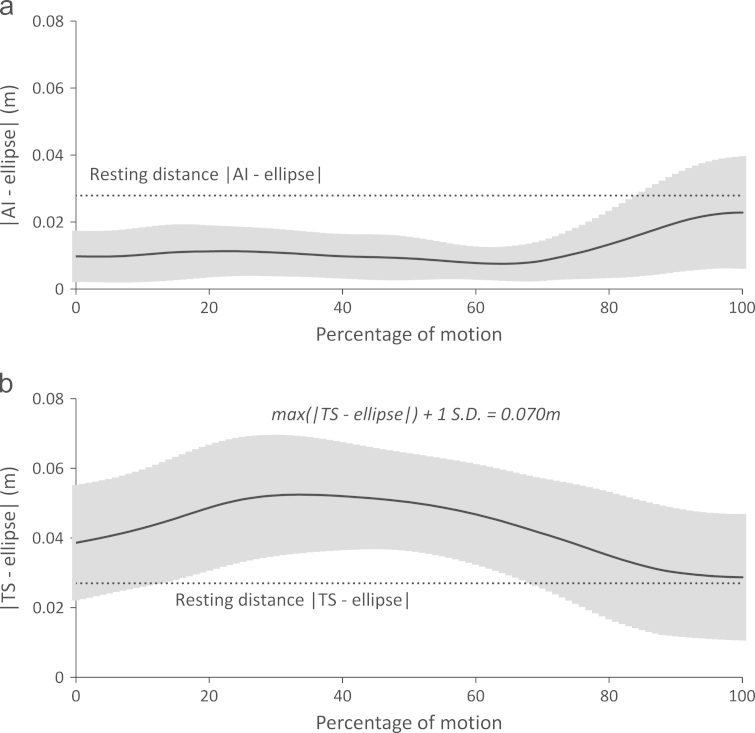
Separation between ellipse representing the STGP and the (a) inferior angle of the scapula and (b) trigonum spinae during the wide motion: using the PCC method of kinematics optimization. The rest position of the two landmarks is shown as the measured separation distances at a position of rest, with the arms by the side and no load on the hands. The grey area represents the positive and negative standard deviation around the mean (black line). The separations presented are the mean values during the wide pull-ups, but should be considered representative of those seen in the two other pull-up tasks.

The distance between the bony landmarks on the scapula medial border and the ellipse representing the STGP are of a similar order to the landmarks׳ resting distances, although the TS distance can be up to twice the resting distance (Fig. 4).

## 4 Discussion

The PCC optimisation and novel thorax scaling provide continuous solutions in all pull-up trials. The FCC method allows a continuous solution in less than 25% of trials (Table 2). The errors compared to measured kinematics are substantially and significantly (*p*<0.001) reduced with the PCC optimisation and scaling correction factor, when compared to the FCC method. The errors only fall within measurement errors (at the 95% C.I.) with the PCC method (Fig. 2, Fig. 3).

The FCC method facilitates a comparison with modelling standards (Nikooyan et al., 2011), but some models are homogeneously scaled before optimisation with the FCC method (e.g., Charlton, 2003). Homogeneous scaling of all model segments (including the thorax) with the constraints of the FCC method (Table 1) improved solution continuity and accuracy relative to the FCC method, as previously observed (Bolsterlee et al., 2013). However, the errors were larger than with the PCC method and significantly larger than measurement errors (Fig. 2, Fig. 3 caption text). Additionally, 37% of trials were non-continuous with a constant conoid length and 3% non-continuous without a constant length.

The PCC optimisation shows large separations of the scapula medial border from the thorax (maximum mean 5.3 cm; Fig. 4). The presented wide pull-up is representative of the other pull-up׳s pattern and magnitude of separation. Separation primarily occurs at TS, with AI generally staying below the rest separation distance. This pattern is likely to be physiological due to the loading condition; the scapula is levered backwards (Fig. 1), increasing separation of TS and pressing AI against the thorax. Also, the large hand forces will cause the arms to translate superiorly, potentially lifting TS above the thorax surface. Cadaver values for rest separation (TS: 3.72 cm and AI: 2.63 cm; Klein Breteler et al., 1999) are similar to the mean values found here (Fig. 4). Although reasonable explanations for the large separations, the true values are not known in highly loaded overhead activities like pull-ups. Further work could utilise bi-planar fluoroscopy (Bey et al., 2006) or open-MRI (Sahara et al., 2007) in quantifying these separations. A maximum separation bounding may be sensible, particularly using a weighted optimisation approach (Bolsterlee et al., 2013).

A study using an optimisation approach to restrict the scapula separation (Bolsterlee et al., 2013) found smaller separation (−1 to 1.5 cm) during a quasi-static movement, where separation is expected to be small compared to pull-ups. The errors from measured scapula rotations are of a similar order, although the range is smaller with the PCC methods here. The range of errors from measured scapula rotations with the FCC method is similar between the two studies.

Future work could utilise non-homogeneous bone morphing techniques (Kaptein and van der Helm, 2004, Yang et al., 2008). The large variation in the externally palpatable landmarks makes scaling difficult (MacGillivray et al., 1998, Pappas et al., 2006). Recent work used coracoid process palpation to improve this with a significant improvement in biceps origin scaling (Bolsterlee and Zadpoor, 2013).

The kinematics optimisation objective function is a convex programming problem, but with the constraints taken into account the feasible regions of both methods are non-convex. Testing five hundred variations of the first frame joint angles increased the likelihood of finding a global minimum within the physiologically feasible region. The mean first-order optimality measure of 3.8×10^−5^ for the FCC method shows that KKT points are generally reached, and a jump in the kinematics data corresponded to a within-trial maximum first-order-optimality measure above 1×10^−3^ on only two occasions, with values of 1.1×10^−3^ and 1.2×10^−3^ observed. These values are low and imply KKT values were reached, suggesting that the jumps observed are not the results of the optimiser failing to reach a minimum.

The constraint that the conoid ligament is a fixed length is shown to have the largest effect on the solution continuity (Table 2) and the errors relative to measured rotations (Fig. 2, Fig. 3). This constraint was originally designed to define the clavicle axial rotation, given the ligament׳s high stiffness and large moment arm around the clavicle long-axis (van der Helm, 1994). Model sensitivity to clavicle axial rotation is probably small compared with sensitivity to the scapula position in relation to the thorax (determined by clavicle protraction and elevation). The ligament is probably not a fixed length during arm elevation tasks (Izadpanah et al., 2012, Seo et al., 2012) and the ligament attachments may be inconsistent between subjects. Therefore, given the serious detrimental effect that the constraint has on solution continuity and the large and significant effects on accuracy, it is recommended that the constraint not be used in kinematics optimisation.

Soft-constraint of the conoid ligament length is not considered appropriate because of high model sensitivity to a parameter that cannot be accurately determined due to inter-subject anatomical variations and a lack of non-invasive clavicle axial rotation measurement techniques. Inclusion in later stages of musculoskeletal modelling (e.g. load-sharing optimisation) is, however, likely to be required - probably with constant or regression-predicted strain values.

The thorax scaling correction factor had little effect on solution continuity (Table 2). The effects on errors compared to measured rotations are generally significant (*p*<0.01) but small (Fig. 2, Fig. 3). Therefore the limiting factor for solution continuity is seemingly the conoid ligament constraint. Standard thorax scaling uses thorax width, depth and height for scaling (Charlton, 2003), with no consideration of the area under the scapula. RMS errors of 5.9 mm have been found between thorax surface points and a fitted ellipse (Bolsterlee et al., 2013). For future work other shapes may be more appropriate.

A combination of the methods discussed is probably optimal: a more complex shape representing the thorax, scaling taking into account the area under the scapula, excluding the conoid ligament constraint from kinematics optimisation and optimisation of separation constraints based on in vivo measures accounting for the separations around the superior thorax.

## Conflict of interest statement

The authors declare that there are no financial or personal relationships with people or organisations that have inappropriately influenced this work.

## References

[bib1] Bey M.J., Zauel R., Brock S.K., Tashman S. (2006). Validation of a new model-based tracking technique for measuring three-dimensional, in vivo glenohumeral joint kinematics. J. Biomech. Eng..

[bib2] Bolsterlee B., Veeger H.E., van der Helm F.C. (2013). Modelling clavicular and scapular kinematics: from measurement to simulation. Med. Biol. Eng. Comput..

[bib3] Bolsterlee B., Veeger H.E.J., van der Helm F.C.T. (2012).

[bib4] Bolsterlee B., Zadpoor A.A. (2013). Transformation methods for estimation of subject-specific scapular muscle attachment sites. Comput. Methods Biomech. Biomed. Eng..

[bib5] Charlton I.W. (2003).

[bib6] Charlton I.W., Johnson G.R. (2006). A model for the prediction of the forces at the glenohumeral joint. Proc. Inst. Mech. Eng. Part H: J. Eng. Med..

[bib7] Dickerson C.R., Chaffin D.B., Hughes R.E. (2007). A mathematical musculoskeletal shoulder model for proactive ergonomic analysis. Comput. Methods Biomech. Biomed. Eng..

[bib8] Harris R.I., Vu D.H., Sonnabend D.H., Goldberg J.A., Walsh W.R. (2001). Anatomic variance of the coracoclavicular ligaments. J. Shoulder Elb. Surg..

[bib10] Izadpanah K., Weitzel E., Honal M., Winterer J., Vicari M., Maier D., Jaeger M., Kotter E., Hennig J., Weigel M., Sudkamp N.P. (2012). in vivo analysis of coracoclavicular ligament kinematics during shoulder abduction. Am. J. Sports Med..

[bib11] Kaptein B.L., van der Helm F.C. (2004). Estimating muscle attachment contours by transforming geometrical bone models. J. Biomech..

[bib12] Karduna A.R., McClure P.W., Michener L.A., Sennett B. (2001). Dynamic measurements of three-dimensional scapular kinematics: a validation study. J. Biomech. Eng..

[bib13] Klein Breteler M.D., Spoor C.W., Van der Helm F.C. (1999). Measuring muscle and joint geometry parameters of a shoulder for modeling purposes. J. Biomech..

[bib14] MacGillivray J.D., Fealy S., Potter H.G., O׳Brien S.J. (1998). Multiplanar analysis of acromion morphology. Am. J. Sports Med..

[bib15] Martelli, S., Veeger, H.E., Van der Helm, F.C., 2008. Scaling of a shoulder musculoskeletal model does not lead to significant improvements. In: Proceedings of the 7th Conference of the International Shoulder Group. University of Bologna, Italy.

[bib16] Nikooyan A.A., Veeger H.E., Chadwick E.K., Praagman M., van der Helm F.C. (2011). Development of a comprehensive musculoskeletal model of the shoulder and elbow. Med. Biol. Eng. Comput..

[bib17] Nikooyan A.A., Veeger H.E., Westerhoff P., Graichen F., Bergmann G., van der Helm F.C. (2010). Validation of the Delft Shoulder and Elbow Model using in-vivo glenohumeral joint contact forces. J. Biomech..

[bib18] Pappas G.P., Blemker S.S., Beaulieu C.F., McAdams T.R., Whalen S.T., Gold G.E. (2006). in vivo anatomy of the Neer and Hawkins sign positions for shoulder impingement. J. Shoulder Elb. Surg..

[bib19] Prinold J.A., Shaheen A.F., Bull A.M. (2011). Skin-fixed scapula trackers: a comparison of two dynamic methods across a range of calibration positions. J. Biomech..

[bib20] Rios C.G., Arciero R.A., Mazzocca A.D. (2007). Anatomy of the clavicle and coracoid process for reconstruction of the coracoclavicular ligaments. Am. J. Sports Med..

[bib21] Sahara W., Sugamoto K., Murai M., Yoshikawa H. (2007). Three-dimensional clavicular and acromioclavicular rotations during arm abduction using vertically open MRI. J. Orthop. Res..

[bib22] Seo Y.J., Yoo Y.S., Noh K.C., Song S.Y., Lee Y.B., Kim H.J., Kim H.Y. (2012). Dynamic function of coracoclavicular ligament at different shoulder abduction angles: a study using a 3-dimensional finite element model. Arthroscopy.

[bib23] Shaheen A.F., Alexander C.M., Bull A.M. (2011). Effects of attachment position and shoulder orientation during calibration on the accuracy of the acromial tracker. J. Biomech..

[bib24] Shaheen A.F., Alexander C.M., Bull A.M. (2011). Tracking the scapula using the scapula locator with and without feedback from pressure-sensors: a comparative study. J. Biomech..

[bib25] Takase K. (2010). The coracoclavicular ligaments: an anatomic study. Surg. Radiol. Anat..

[bib26] van der Helm F.C.T. (1994). A finite element musculoskeletal model of the shoulder mechanism. J. Biomech..

[bib27] Wu G., Siegler S., Allard P., Kirtley C., Leardini A., Rosenbaum D., Whittle M., D׳Lima D.D., Cristofolini L., Witte H., Schmid O., Stokes I. (2002). ISB recommendation on definitions of joint coordinate system of various joints for the reporting of human joint motion. Part I: ankle, hip, and spine. J. Biomech..

[bib28] Yang Y.M., Rueckert D., Bull A.M.J. (2008). Predicting the shapes of bones at a joint: application to the shoulder. Comput. Methods Biomech. Biomed. Eng..

